# Effect of Finisher Gentlefile brush, XP-endo Finisher and ultrasonic irrigation for the removal of calcium hydroxide paste from root canal

**DOI:** 10.6026/9732063002001052

**Published:** 2024-09-30

**Authors:** Ojaswini Pawar, Gurudutt Nayak, Philip Pradeep, Vivekananda Pai A.R., Drisya D Nambiar, Ramanathan Ravi, Sonam Bohra

**Affiliations:** 1 Department of Conservative Dentistry and Endodontics, Mansarovar Dental College, Bhopal, Madhya Pradesh, India; 2 Department of Conservative Dentistry and Endodontics, Faculty of Dentistry, Manipal University College Malaysia, Melaka, Malaysia

**Keywords:** Calcium hydroxide, Finisher Gentlefile Brush, Passive Ultrasonic Irrigation, XP-endo Finisher

## Abstract

Eliminating residual calcium hydroxide [Ca(OH)_2_] intracanal medicament from the walls of a root canal presents a persistent obstacle that can impede the establishment of a proper fluid-tight seal during obturation. The effective removal of these medicaments from the canal walls has consistently posed a significant challenge. Consequently, several systems have been developed and assessed in order to address this issue. Therefore, it is of interest to compare the efficacy of Finisher Gentlefile Brush, XP-endo Finisher and Passive Ultrasonic Irrigation (PUI) on the removal of an oil-based Ca(OH)_2_ paste. 60 human mandibular extracted premolars were selected and underwent preparation with Pro Taper Gold rotary file system till size F5. The canals were dried and filled with Metapex and stored for a week. Further, the specimens were divided into 3 groups depending upon the Metapex removal protocols, namely, Finisher Gentlefile Brush, XP-endo Finisher, and PUI. Afterward, the specimens were sectioned buccolingually. Evaluation of remnants was done with a scoring system under a dental microscope at 25x magnification. The data was analyzed using the Chi-square test (p<.05). With the exception of PUI, all the methods demonstrated significantly better performance, with Finisher Gentlefile Brush being the most effective across all canal thirds (p<.05). While none of the methods achieved complete cleanliness, Finisher Gentlefile Brush exhibited exceptional results compared to the other two systems employed.

## Background:

The predictable and successful aftermath following endodontic therapy is governed by the abolition of microbiota and associated waste from the root canal system. However, the current shaping and cleaning procedures fall short of meeting the criteria for a thoroughly disinfected canal [1]. As a result, the use of intracanal medicaments becomes crucial in overcoming such limitations [2]. One commonly utilized intracanal medication is Ca(OH)_2_, which is widely favoured and accepted by dental healthcare practitioners [3]. One of the primary factors contributing to its preference is its mechanism of action, involving the ionic dissociation into hydroxyl and calcium ions. These ions effectively penetrate the dentinal tubules, leading to the eradication of microorganisms. Additionally, Ca(OH)_2_ exhibits effectiveness against a broad spectrum of endodontic bacteria, possesses tissue-dissolving properties, promotes mineralization and suppresses osteoclastic activity [4]. Various formulations of Ca(OH)_2_ are available, which include aqueous, viscous and oil-based forms depending upon the type of vehicle used. The purpose behind using these vehicles is to improve certain properties such as ease of use, radio-opacity, rate of dissociation of ions in between appointments and ease of removal [5, 6]. In the aqueous-based form, the vehicles used for Ca(OH)_2_ include water, saline, dental anesthetics and Ringer's solution. These vehicles facilitate the rapid release of ions and enhance solubility with tissues. On the other hand, the viscous-based form utilizes vehicles such as glycerine, propylene glycol and polyethylene glycol. These vehicles are water-soluble and have higher molecular weights, resulting in a slower rate of dissociation compared to the aqueous-based forms [6, 7]. Oil-based forms, however, have the lowest solubility and limited diffusion into the tissues. Vehicles used for oil-based forms include olive oil, silicone oil, metacresylacetate, camphor and certain fatty acids [7]. Metapex (META Biomed Co. Ltd, Chungcheongbuk-do, Korea) is a commercially used oil-based Ca(OH)_2_ that consists of iodoform and silicone oil which is available in an injectable form [8].

Complete removal of Ca(OH)_2_ from the root canal walls is essential before obturation to ensure proper adhesion of the sealer and obturating materials to the canal walls. However, several studies have highlighted the difficulty in achieving the complete removal of Ca(OH)_2_ along the canal walls [9, 10, 11, 12, 13, 14-15]. The presence of residual remnants of Ca(OH)_2_ has been shown to impede sealer penetration into dentinal tubules, leading to potential apical leakage. Additionally, it can result in alterations in the physical characteristics and setting of sealers, as well as reduced bond strength [15, 16-17]. The cleansing of the canal walls of the intracanal medicament is accomplished using various tools and methods, of which PUI and the XP-endo Finisher have been found to be the most effective in previous researches [10-14, 18]. PUI operates on a mechanism based on the passive agitation of irrigants inside the root canal. This occurs due to an oscillating ultrasonic tip attached to a device, which on entering the root canal filled with irrigant, causes cavitation and acoustic streaming [19, 20]. Several devices are used for PUI, out of which, most recently, the Ultra-X ultrasonic irrigation device has been proposed [21]. It comprises the Ultra-X ultrasonic activator and three different tips for various purposes. The blue tip is known for its flexibility and is used to activate irrigants in canal curvatures and remove gutta-percha during retreatment. The silver tip is a soft and flexible tip for activating irrigants in challenging curved canals. The gold tip is a powerful titanium tip for retrieving separated instruments from the canal. All these tips are available in sizes 20/0.02 and 25/0.02.

The XP-endo Finisher (FKG Dentaire, La-Chaux-de-Fonds, Switzerland) is an instrument that works on the Max Wire Nickel-Titanium (Ni-Ti) alloy's shape memory concept. With a diameter of ISO 25, it is designed for canals with complex morphology and features a zero-degree taper. When the instrument is rotated, it exhibits remarkable flexibility and can expand up to 100 times its original size, reaching a diameter of 6 mm in the final 10 mm of the file. When the file is cooled to room temperature (20°C), it is initially straight (M-phase or Martensite phase), but when exposed to body temperature (35°C) or inside the root canal, molecular memory causes it to shift to a curve or sickle shape (A-phase or Austenite phase). This shape of the instrument in the A-phase enables it to access the difficult regions of the root canal. As stated by the guidelines of the manufacturer, it facilitates the removal of obturating material during retreatment and medicaments from root canals by guiding the chemical solutions or irrigants in inaccessible areas [12]. While techniques such as PUI and the XP-endo Finisher have shown superiority, the complete removal of Ca(OH)_2_ from root canals remains a challenge, particularly in teeth with oval canals. To address this issue, the introduction of a modified instrument called the Gentlefile system, which includes the innovative Finisher Gentlefile Brush, may offer improved assistance in the complete removal of root canal medicaments. The Gentlefile system is a novel root canal instrumentation system consisting of a cordless handpiece, files for instrumentation of the root canal and Finisher Gentlefile Brush to activate irrigants inside the root canal. The Finisher Gentlefile Brush has six strands of stainless-steel flexible strings that open outward once operated by the handpiece at 6500 rpm. Previous studies have primarily focused on the efficacy of the Finisher Gentlefile Brush for irrigation and the amount of dentin loss [22, 23]. Therefore, it is of interest to evaluate the efficacy of Finisher Gentlefile Brush, XP-endo Finisher and PUI for removing an oil-based Ca(OH)_2_ paste from the root canals.

## Method and Materials:

## Sample size calculation:

The sample size was calculated using the formulae n = n0 / 1 + (n0-1)/N; with a confidence level of 95%, a confidence interval of +/- 5%, and a standard deviation of 0.5, the study's sample size was twenty times the number of participants in each group. This was determined using the Cochran technique.

## Sample selection and specimen standardization:

Based on the radiographic analysis, 60 human mandibular premolars with a single straight root and a patent root canal that were extracted for orthodontic or periodontal reasons were chosen. Teeth with any signs of immature apices, resorption, prior endodontic treatment, caries, calcification, prior restoration, cracks and fractures were excluded from the study. The teeth were scaled with ultrasonics to remove any calculus or soft tissue debris and were placed in 10% formalin solution until use.

## Root canal instrumentation:

Working Length (WL) was done precisely 1 mm short of the length when a #10K file (Dentsply Maillefer, Ballaigues, Switzerland) was visible at the apical foramen when viewed under a microscope (Labomed PRIMA DNT; Labo America Inc., Fremont, CA) at 25x magnification. The ProTaper Gold rotary system (Dentsply Sirona, Ballaigues, Switzerland) was used for canal preparation, following the crown-down technique, up to size F5 (#50/0.05 taper). An endodontic motor (X-Smart; Dentsply Maillefer, Ballaigues, Switzerland) with torque and speed control was utilized according to the manufacturer's instructions. After each instrument change, the root canals were thoroughly irrigated with 2 mL of a 3% sodium hypochlorite (NaOCl) solution (PrevestDenpro Ltd, Jammu, India) using a 30-gauge close-end tip and a double side-port opening needle (RC Twents, Prime Dental Products Pvt. Ltd., Maharashtra, India). After completion of the preparation, a final rinse of the root canal was performed with 5 mL of 17% EDTA solution (Prevest Denpro Ltd, Jammu, India) and 5 mL of normal saline, following which sterile paper points were used to dry the canals.

## Ca(OH)_2_ placement:

Prepared root canals were filled with Metapex using special tips provided by the manufacturer. Complete filling of the root canals was ensured by radiographs taken in mesiodistal and buccolingual directions. The coronal part of the canal was sealed with a provisional filling material (Cavit G; 3M ESPE Dental Products, St Paul, MN). Samples were stored at a temperature of 37°C and at 100% humidity for a week in an incubator to simulate oral conditions during inter-appointment dressing.

## Ca(OH)_2_ removal:

The samples were randomly divided into three experimental groups (n = 20) according to the Ca(OH)_2_ removal method. In all the groups, following the removal of the provisional filling material, a size #15K file was introduced up till the WL to loosen the Ca(OH)_2_ and make room for the irrigating needle to enter. The canals were first irrigated in all the specimens using 5 mL of 3% NaOCl with the needle set at 1 mm from the WL with a flow rate of 5 mL/min.

## Group 1: Finisher Gentlefile Brush:

Following the previous steps, the Finisher Gentlefile Brush, with a tip size of 0.25 mm, was used to agitate the solution for 1 minute at amplitude of 7-8 mm while operating at a speed of 6500 rpm and 1 mm short of the WL. Similarly, the canals were then irrigated with 5 mL of 17% EDTA solution, which was agitated for another 1 minute, as performed previously. This was followed by a final flush of 5 mL of normal saline. One Finisher Gentlefile Brush was used per specimen.

## Group 2: XP-endo Finisher:

A torque-controlled electric endodontic motor with a speed set at 800 rpm and 1 Ncm torque was mounted with a size 0.25 mm XP-endo Finisher file. While the file was still in the plastic tube, it was adjusted up to the WL with a rubber stopper. The file was then chilled with a cold spray (Roeko Endo-Frost spray; Coltene-Whaledent, Langenau, Germany) to make it straight. Then, with a slight lateral movement, the file was extracted from the plastic tube. The XP-endo Finisher file was inserted 1 mm short of the WL and employed in a gradual up-and-down movement with an amplitude of 7-8 mm for 1 minute. Then, the canals were irrigated with 5 mL of 17% EDTA solution and the XP-endo Finisher file was used as described earlier. This was followed by a final flush of 5 mL of normal saline. One XP-endo Finisher file was used per specimen.

## Group 3: PUI:

PUI was performed using an Ultra-X ultrasonic irrigation device and a #25/0.02 Ultra-X tip. The Ultra-X tip was positioned 1 mm short of the WL, followed by activation of the irrigating solution for 1 minute. Then the canals were irrigated with 5 mL of 17% EDTA solution and further agitated with an Ultra-X tip for 1 minute. Each specimen was then cleaned with 5 mL of normal saline. One Ultra-X tip was used for three samples.

## Assessment of the remaining amount of Ca(OH)_2_:

**Figure 1:**
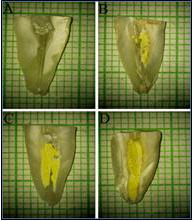
Depicts scores from 0-3 representing the remaining amount of Ca(OH)_2_ in the root canal walls when observed and evaluated from images obtained with a dental operating microscope.

Following irrigation, absorbent paper points (Dentsply Sirona, Ballaigues, Switzerland) were used to dry the root canals. To enable the splitting of the root for exposing the root canal, on the buccal and lingual sides, two grooves were created in a longitudinal fashion. This was done at the maximum buccolingual width of the root, which was achieved using a diamond disc mounted on a handpiece under copious water cooling. Then with utmost precaution, the tooth was finally split into two longitudinal halves with the help of a chisel and mallet. The appropriate half of each root with a visible semi-canal lumen having higher Ca(OH)_2_ remnants was selected. The samples were placed on a mm2 graph paper to calibrate the coronal, middle and apical thirds of the canal space. Images were captured with the digital camera (Canon EOS1300D, Canon Inc., Taiwan) connected to a dental operating microscope at 25x magnification. To prevent the examiners from identifying the specimen, the images were coded. Two calibrated examiners not aware of the experimental groups scored the images for the remaining amount of Ca(OH)_2_ in the canal based on the classification provided by van der Sluis *et al.* [19], where a score "0" denotes root canal surface free of Ca(OH)_2_, "1" denotes less than half of the root canal surface filled with Ca(OH)_2_, "2" denotes more than half of the root canal surface filled with Ca(OH)_2_, and "3" denotes root canal surface completely filled with Ca(OH)_2_ (Figure 1). Then in all the canal thirds of the chosen half of the samples, remnants of Ca(OH)_2_ were assessed, scored and recorded separately. Any disagreements between the examiners were reassessed with a joint discussion to reach an agreement on the scores.

## Statistical analysis:

All the gathered data was analyzed using SPSS software for Windows, version 26.0 (IBM SPSS Inc., Chicago, IL, USA). The efficacy of various Ca(OH)_2_ removal methods was expressed in percentage. Utilizing the Chi-square test, categorical variables were compared. The level of significance was set at p-value < .05. Inter-examiner agreement was assessed using the Cohen kappa coefficient.

## Results:

**Figure 2:**
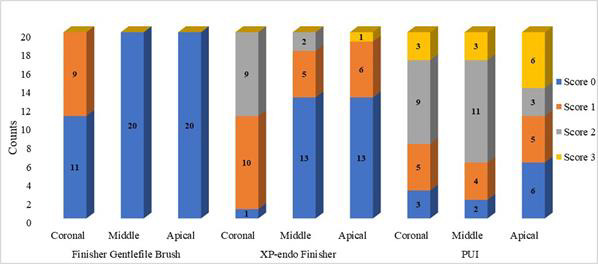
Bar graph depicting score distribution in the canal thirds with respect to the three devices used.

The inter-examiner agreement showed strong agreement between the examiners with a Cohen kappa value of 0.819. Figure 2 shows the distribution of score in the coronal, middle and apical third of the canals between the three types of devices used. The results showed that neither of the groups tested removed Ca(OH)_2_ entirely from the root canal. The intergroup comparison in the coronal, middle, and apical third showed that the remaining amount of Ca(OH)_2_ was found to differ significantly between the groups (p<.05) (Table 1). The Finisher Gentlefile Brush group showed the highest number of samples with complete removal of Ca(OH)_2_ in all the canal thirds compared to the XP-endo Finisher group and PUI group (p<.05). The XP-endo Finisher group was significantly cleaner from Ca(OH)_2_ in the apical and middle third of the canal in contrast to PUI group (p<.05) (Table 1). The intra-group comparison showed significant differences in scores for the Finisher Gentlefile Brush group and XP-endo Finisher group for removal of Ca(OH)_2_ in the coronal, middle and apical third of the root canal (p<.05). However, there was no statistically significant difference seen in PUI group (p>.05) (Table 1).

## Discussion:

Root canal asepsis is of prime importance in achieving a successful outcome in endodontic treatment. To fulfill this criteria use of intracanal medicament is one of the regimens followed by most clinicians. Their use as intracanal dressings plays a major role due to their bacteriostatic nature preventing bacterial proliferation and acting as a physiochemical barrier against the spread of infection in the root canals [24]. The most preferable and highly recommended intracanal medicament amongst clinicians is Ca(OH)_2_, which is available in various formulations and viscosities and displays a well-recognized antibacterial activity [25]. After its use inside the root canal, its thorough eradication is also mandatory to achieve a fluid-tight seal during obturation because it tends to have good retention to the dentinal tubules, blocking the adhesion of sealer to the root canal dentin and ultimately leading to microbial percolation [26].

The vehicles used to carry the Ca(OH)_2_ medicament play a vital role in its removal from the root canal. Studies done by Nandini *et al.* [8] and Turkaydin *et al.* [11] proved that water-based Ca(OH)_2_ paste was easily removed compared to oil-based paste, irrespective of the removal methods used. Removal of Metapex has always been challenging due to its iodoform and silicone oil content. The property of silicone oil to resist dissolution in water is what causes Metapex to retain itself to the root canal walls [8]. Therefore, in this study, Metapex, an oil-based Ca(OH)_2_ was used as the intracanal medicament to assess its retrievability from the root canals. Previous studies have employed multiple techniques for the removal of Metapex, including hand instrumentation, rotary instrumentation, conventional syringe irrigation, manual dynamic agitation, ultrasonic agitation, sonic agitation, canal brush, and laser activation. These techniques were often used individually or in combination with various irrigants, such as 17% EDTA, 0.2% chitosan, 10% maleic acid, 10% citric acid and 3% NaOCl [8, 25, 27- 28, 29]. Among all these methods PUI [8, 10, 14, 21, 30] and XP-endo Finisher [10, 12, 13] have been shown to be the most efficacious but did not show complete eradication of Ca(OH)_2_.

In this study, PUI and XP-endo Finisher were compared with Finisher Gentlefile Brush for the removal of Metapex from the root canals. PUI has been the standard in most studies for comparing Ca(OH)_2_ removal with other systems used. Various devices for PUI are available in the market. Ultra-X ultrasonic irrigation device is a cordless system that works at a frequency of 45 kHz and aims to clean and clear complex and intricate areas of the root canal system because of its capacity to transmit acoustic energy to the irrigant inside the canal that originates from the oscillating ultrasonic instrument [31, 32]. It has a particular pattern of forming streams in an apico-coronal direction because of cavitation and acoustic streaming [33]. This type of irrigant agitation makes the irregular and inaccessible areas of the root canal susceptible to Ca(OH)_2_ eradication, proving its removal efficacy [34]. This study observed that PUI could not remove Metapex completely from any of the canal thirds, and chunks of medicament were seen after the samples were sectioned longitudinally and observed under magnification. The results showed no significant difference between all the canal thirds and the specimens showed the highest remnants of medicament compared to the other two systems used. This could be because PUI involves creating explosion and implosion of bubbles to energize the irrigant without actual contact of the tip with the canal wall limiting its potential to effectively remove the Ca(OH)_2_ [35]. These findings are consistent with the studies that have been previously performed where PUI was compared with other mechanical devices [11, 13, 18, 35].

Apart from PUI the XP-endo Finisher file is the preferred instrument for comparing the efficacy of other methods in removing Ca(OH)_2_. This single-file system demonstrates enhanced Ca(OH)_2_ removal, even in intricate areas of the root canal, surpassing the performance of systems like PUI. These findings align with the results of this study, as the XP-endo Finisher file exhibited significantly better performance than PUI in the apical third of the canal. This can be attributed to the superior design and properties of the XP-endo Finisher file, which physically contacts all parts of the canal, including curvatures and oval areas, in contrast to the non-contact mode of action of PUI [35, 36]. Multiple studies have demonstrated that the usage of XP-endo Finisher is either superior or equally effective compared to PUI in removing intracanal medicament [12, 35, 37]. The effectiveness of XP-endo Finisher, particularly in the apical third of the root canal, was observed in investigations conducted by Denna *et al.* [12] and Kfir *et al.* [37], aligning with the findings of the current study. The incomplete removal of Ca(OH)_2_ from the middle and coronal thirds of the canal can be attributed to the shape of the canal. As we progress from the apical to the coronal third, the canal gradually becomes more oval, especially in mandibular premolars, thereby reducing the ability of any instrument to access and touch all the surfaces of the canal [38]. Another reason for its reduced efficacy is its intended use as a finisher file which is meant to clean previously shaped root canals [37]. Though XP-endo Finisher performed superiorly in the apical third compared to PUI, it still lacked complete cleanliness in the middle and coronal third and showed similar efficacy. The Gentlefile is a unique system for rotary instrumentation that offers an automated handpiece for shaping and irrigating root canals. The unique mechanism and design of the Finisher Gentlefile Brush demonstrated promising results in this study, as it not only completely removed Metapex from the apical and middle thirds of the root canal but also exhibited superior removal in the coronal third when compared to XP-endo Finisher and PUI. It is important to note that no previous studies have evaluated the efficacy of the Finisher Gentlefile Brush in medicament removal, making the positive outcome of this study particularly significant [22, 23]. The Finisher Gentlefile Brush, with six strands of stainless-steel flexible wires that expands out on activation, contacts the canal walls exceptionally, even in the middle and coronal third. Further, the instrument activates the irrigant through centrifugal movement, creating a whirlpool effect. This ensures that the irrigant comes in contact with the canal walls throughout the space, thus removing the intracanal medicament. The efficiency of medicament removal is enhanced by the fact that the instrument rotates at 6500 rpm, which leads to the increased number of times that the strands of the Finisher Gentlefile Brush physically come in contact with the canal wall and enhancing the activation of the irrigant which substantiates the results achieved. To evaluate the residual amount of intracanal medicament, the scoring system, which is the most preferred and common method was undertaken. Other methods including volumetric analysis with micro-CT, scanning electron microscope, and the use of softwares to calculate the area of residual intracanal medicament [27, 28, 29-30,33, 34] could have been used for more precise data. The favorable result of Finisher Gentlefile Brush achieved in the study opens a new avenue for evaluation of intracanal medicament removal from resorptive cavities which is further challenging due to its irregular intricacies. Studies have been done where intracanal medicament was removed by the use of chemicals in conjunction with mechanical aids [8,27]. Hence, the use of these chemicals along with Finisher Gentlefile Brush may prove to be even more efficient and warrants future research.

## Conclusion:

Data shows that the Finisher Gentlefile Brush performed strikingly well and showed promising results compared to XP-endo Finisher and PUI in terms of canal cleanliness, irrespective of the canal morphology or the type of medicament used.

## Figures and Tables

**Table 1:** Distribution of percentage of Ca(OH)_2_ removal scores for each of the groups tested.

**Group**	**Canal 3** ^**rd**^	**0 (%)**	**1 (%)**	**2 (%)**	**3 (%)**
Finisher	Coronal^a^	11(55.0)	9(45.0)	0(0.0)	0(0.0)
Gentlefile	Middle^b^	20(100.0)	0(0.0)	0(0.0)	0(0.0)
Brush^A^	Apical^c^	20(100.0)	0(0.0)	0(0.0)	0(0.0)
XP-endo	Coronal^a^	1(5.0)	10(50.0)	9(45.0)	0(0.0)
Finisher^B^	Middle^b^	13(65.0)	5(25.0)	2(10.0)	0(0.0)
	Apical^c^	13(65.0)	6(30.0)	0(0.0)	1(5.0)
PUI^C^	Coronal^a^	3(15.0)	5(25.0)	9(45.0)	3(15.0)
	Middle^a^	2(10.0)	4(20.0)	11(55.0)	3(15.0)
	Apical^a^	6(30.0)	5(25.0)	3(15.0)	6(30.0)
Groups and canal 3rds with different superscript letters were statistically significant at p<.05
